# Round the Hospitals

**Published:** 1917-05-12

**Authors:** 


### ROUND THE HOSPITALS.

The College of Nursing and British-trained nurses throughout the Empire owe a debt of grateful recognition to the British Women's Hospital Committee and all connected with it, who are raising the Nation's Fund for Nurses, as a tribute from the British Empire to British nurses. In The Hospital, March 24, page 502, we exposed the humbug of the Lady's claim that "to raise an Endowment Fund for the Royal College of British Nursing has aroused the self-respecting element in the nursing profession." It is not necessary, therefore, to repeat the exposure, because her supporters are again trying to confuse the issue by this absurd cry which is confined to the followers of the Society for the State Registration of Trained Nurses, a relatively small body, as we have shown, in which the great bulk of the nursing profession take no interest. The Endowment Fund for the College to be raised by the British Women's Hospital should approach, if it does not exceed, £250,000. This is a considerable sum in one sense, though not as a tribute from the British Empire to British nurses. We hope that the subscription list will include many wealthy men and women, for, after all, wealth without the aid of physicians and nurses cannot buy adequate treatment and attendance for its possessors in the day of sickness or accident. That this is recognised by the wealthy is demonstrated by an intimation we have received this week of a contemplated gift of £25,000 to aid working nurses in this country in their declining years. As a matter of business to be successful the College Endowment Fund must then secure many large gifts, but recognising there are very few people that pass through life without owing a debt of gratitude to nurses, we share Mr. Stanley's hope that the Nation's Fund for Nurses will constitute in its specially representative character a real tribute of gratitude from the multitude of all classes of the community who have had much benefit themselves from the ministrations of the nurses.

---

The founding of the College of Nursing and the opening of its Register is likely to reveal with telling force the disabilities and avoidable disaster which have attended and still attend a large number of eligible women who, wishing to enter the nursing profession, fail to make it their business to learn the facts, before entering the service of Institutions or Private Homes, which profess to train probationers without having either the facilities or the means to do so. There are, to the disgrace of those who are responsible for the fraud upon innocent women seeking nurse-training, a large and, we fear, increasing number of establishments of the kind, who, by promising to train and certificate young women who enter their service, obtain the assistance they require for their own purposes, frequently for a paltry payment or for no payment at all. Anyone who consults "How to Become a Nurse," being a Guide to Training for the Nursing Profession (The Scientific Press, Ltd., 28 Southampton Street, Strand, W.C. 2), might escape the pitfalls which have been the undoing of a multitude of women in these islands, who have missed their way by not entering in the first instance an approved Nurse-training School and have so spoilt or ruined their career.

---

Unfortunately there are a large number of young women still who have had experience in Nursing Homes or worked in several small hospitals for a few months at a time, or have even served for three years in unrecognised workhouse sick-wards, or in a cottage hospital of some twenty beds, who, despite devoted and continuous hard work, find themselves ineligible for Registration with the College of Nursing. The Local Government Board owe it to their reputation as an act of justice to women employees under the Poor Law to take steps to make it plain that service as assistant-nurses in unrecognised workhouse sick wards does not constitute nurse-training nor qualify them for a certificate, which, at the expiration of three years, will make them eligible to have their names on the Register. Cottage hospital authorities throughout the country, too, are to blame which take so-called probationer nurses and retain their services for a lengthened period, knowing they cannot grant a certificate or give adequate training at the cottage hospital without making these facts clear to the young women entering their service and making arrangements with some county or other hospital, whereby they can be passed on from the cottage hospital to a recognised institution where they will receive adequate training and a certificate qualifying for the College Register at the end of three years.

---

We hope the Hon. Arthur Stanley will call Lord Rhondda's attention to the injury done through the Poor Law to many innocent women in this matter, for then a speedy stop will no doubt be put to the existing evils. The exorbitant claims advanced by the National Poor-Law Officers' Association in regard to the numbers of nurses,

**ROUND THE HOSPITALS—(continued).**

so-called, under the Poor Law will be reduced then to a figure more in accordance with the facts than its representatives have heretofore claimed. We may add that every woman victim of the evils here referred to, who is still of a suitable age, should make it her business to seek admission to a recognised training-school and enter herself as a probationer quickly. We hope that matrons in charge of training-schools, who are familiar with the facts, will consort measures to help these innocent victims by facilitating their admission to training, and that the College Council will take the matter in hand and issue a public warning to the nation and women workers throughout the country, for that would be the most effective way of reducing to a minimum for the future the abuses here dealt with.

---

THE *Gazette* of the 4th instant, under Army Honours, announced: "The following ladies are awarded the decoration of the Royal Red Cross in recognition of their valuable services in connection with the war: *Royal Red Cross, First Class*—Miss Eva Charlotte Ellis Luckes, chief matron, London Hospital. *Royal Red Cross, Second Class*—Miss Alice Mead, sister, Convalescent Hospital for Officers, Osborne." We have on more than one occasion recently urged the justice and importance of giving recognition through honours to many devoted workers in the Homeland since the outbreak of war. Miss Luckes has held the responsible post of matron of the London Hospital for thirty-seven years, and it is fitting and just that she should have the Royal Red Cross, First Class, announced in the *Gazette*, in which her name is the only one mentioned as a recipient of this honour. The changes which have come over the hospital and nursing world since Miss Luckes first came from Manchester to Whitechapel to take charge of the London Hospital are multifarious and marvellous. The growth of the London Hospital has proceeded until the bed accommodation it contains is higher than that of any other Metropolitan hospital. This fact testifies in a line to the great increase in the responsibility and work which must have fallen upon the chief matron of the London Hospital during the last thirty-seven years. Miss Luckes, we are glad to know, has recovered from the very serious illness which seized her some months ago, and we wish her the best of health and all that she most desires during the remainder of her working days and throughout her life.

---

THE Council of Queen Victoria's Jubilee Institute were notified, at their meeting on May 2, of the resignation of Miss Amy Hughes of the post of General Superintendent of the Institute, which was accepted by the Council with very great regret. Miss Hughes's connection with district nursing dates from the year 1885, when she left St. Thomas's Hospital to join the Metropolitan and National Association in Bloomsbury. She inaugurated district nursing in the Metropolitan areas of Kensington, Chelsea, and Westminster, and was placed on the Queen's Roll in 1891. With the exception of six years, occupied in nursing work unconnected with the Institute, Miss Hughes has been engaged in district work from 1885 until the present time. In 1902 she was appointed Superintendent of County Nursing Associations. There were at that time five of these affiliated to the Q.V.J.I. The number is now twenty-six. To the greatly increased public interest in the work of district nurses and to the wide extension of their influence as—in Miss Nightingale's phrase—Health Missionaries, the ability and enthusiasm of Miss Hughes has conspicuously contributed during the twelve years she has held the important position of General Superintendent to the Institute. The position of General Superintendent of Queen Victoria's Jubilee Institute for Nurses is one of vast importance to the people and great responsibility for the lady who occupies it. Miss Hughes has shown by her devotion and marked ability how far-reaching is the influence of the General Superintendent of this Institute and how full of benefit to the poorer households throughout the country a wise and capable administrator can make the Institute, its organisation and resources. We are therefore grateful to know that the Council have expressed the wish that Miss Hughes shall continue her connection with the Institute by representing it on the committees and at meetings of certain nursing and other institutions whose objects render collaboration with the Institute desirable. It will be a satisfaction to her many friends, as well as to the Council of the Q.V.J.I., that, "in a position of greater freedom and less responsibility," her connection with district nursing will be maintained. It has been noticeable in recent years how great was the strain under which Miss Hughes was labouring. It was time that that strain should be removed, and we hope that with restored health and strength she may have before her many years full of benefit to the nursing profession and the people, accompanied by sound health and much pleasurable and fruitful occupation for herself.

---

THE Service of Thanksgiving to be held at Westminster Abbey on Ascension Day, May 17, for the work of the Red Cross has excited widespread interest amongst all classes of people. For those of us who are continuously associated with work for the sick, this service has a special significance and importance, and we have little doubt that the congregation at the Abbey on the 17th instant will probably exceed in number almost that present at any service that has ever been held within its walls. No request for tickets or inquiries can be dealt with by the clergy of the Abbey, and all applications for tickets and all inquiries should be addressed to Mr. E. P. Vaughan-Morgan, Room 24, 83 Pall Mall, S.W. 1.

For "Food in Wartime," see p. 118; "Patriotism of the Nurse-Training Schools," see p. 119; "Presentations to Miss Herbert" and "Matrons' and Sisters' Appointments" see p. 120.

